# Remote Monitoring App for Endocrine Therapy Adherence Among Patients With Early-Stage Breast Cancer: A Randomized Clinical Trial

**DOI:** 10.1001/jamanetworkopen.2024.17873

**Published:** 2024-06-27

**Authors:** Ilana Graetz, Xin Hu, Mehmet Kocak, Rebecca A. Krukowski, Janeane N. Anderson, Teresa M. Waters, Andrea N. Curry, Andrew Robles, Andrew Paladino, Edward Stepanski, Gregory A. Vidal, Lee S. Schwartzberg

**Affiliations:** 1 Department of Health Policy and Management, Rollins School of Public Health, Emory University, Atlanta, Georgia; 2 Department of Public Health Sciences, University of Virginia School of Medicine, Charlottesville; 3 International School of Medicine, Istanbul Medipol University, Istanbul, Turkey; 4 Department of Community and Population Health, College of Nursing, University of Tennessee Health Science Center, Memphis; 5 School of Public Health, Augusta University, Augusta, Georgia; 6 West Cancer Center and Research Institute, Germantown, Tennessee; 7 Sidney Kimmel Cancer Center, Jefferson University, Philadelphia, Pennsylvania; 8 College of Medicine, University of Tennessee Health Science Center, Memphis; 9 Ovation, Cambridge, Massachusetts; 10 Pennington Cancer Institute, Renown Health, Reno, Nevada; 11 Department of Medicine, University of Nevada, Reno

## Abstract

**Importance:**

Adjuvant endocrine therapy (AET) use among women with early-stage, hormone receptor–positive breast cancer reduces the risk of cancer recurrence, but its adverse symptoms contribute to lower adherence.

**Objective:**

To test whether remote monitoring of symptoms and treatment adherence with or without tailored text messages improves outcomes among women with breast cancer who are prescribed AET.

**Design, Setting, and Participants:**

This nonblinded, randomized clinical trial (RCT) following intention-to-treat principles included English-speaking women with early-stage breast cancer prescribed AET at a large cancer center with 14 clinics across 3 states from November 15, 2018, to June 11, 2021. All participants had a mobile device with a data plan and an email address and were asked to use an electronic pillbox to monitor AET adherence and to complete surveys at enrollment and 1 year.

**Interventions:**

Participants were randomized into 3 groups: (1) an app group, in which participants received instructions for and access to the study adherence and symptom monitoring app for 6 months; (2) an app plus feedback group, in which participants received additional weekly text messages about managing symptoms, adherence, and communication; or (3) an enhanced usual care (EUC) group. App-reported missed doses, increases in symptoms, and occurrence of severe symptoms triggered follow-ups from the oncology team.

**Main Outcomes and Measures:**

The primary outcome was 1-year, electronic pillbox–captured AET adherence. Secondary outcomes included symptom management abstracted from the medical record, as well as patient-reported health care utilization, symptom burden, quality of life, physician communication, and self-efficacy for managing symptoms.

**Results:**

Among 304 female participants randomized (app group, 98; app plus feedback group, 102; EUC group, 104), the mean (SD) age was 58.6 (10.8) years (median, 60 years; range, 31-83 years), and 60 (19.7%) had an educational level of high school diploma or less. The study completion rate was 87.5% (266 participants). There were no statistically significant differences by treatment group in AET adherence (primary outcome): 76.6% for EUC, 73.4% for the app group (difference vs EUC, −3.3%; 95% CI, −11.4% to 4.9%; *P* = .43), and 70.9% for the app plus feedback group (difference vs EUC, −5.7%; 95% CI, −13.8% to 2.4%; *P* = .17). At the 1-year follow-up, app plus feedback participants had fewer total health care encounters (adjusted difference, −1.23; 95% CI, −2.03 to −0.43; *P* = .003), including high-cost encounters (adjusted difference, −0.40; 95% CI, −0.67 to −0.14; *P* = .003), and office visits (adjusted difference, −0.82; 95% CI, −1.54 to −0.09; *P* = .03) over the previous 6 months compared with EUC participants.

**Conclusions and Relevance:**

This RCT found that a remote monitoring app with alerts to the patient’s care team and tailored text messages to patients did not improve AET adherence among women with early-stage breast cancer; however, it reduced overall and high-cost health care encounters and office visits without affecting quality of life.

**Trial Registration:**

ClinicalTrials.gov Identifier: NCT03592771

## Key Points

**Question:**

Does a remote symptom and medication adherence monitoring app with or without tailored text messages improve outcomes for women with early-stage breast cancer starting adjuvant endocrine therapy (AET)?

**Findings:**

In this randomized clinical trial of 304 women, a remote monitoring app combined with text messages did not improve AET adherence in the first year (the primary outcome) but increased symptom management and reduced overall and high-cost health care encounters and office visits (secondary outcomes). The app alone did not significantly affect outcomes.

**Meaning:**

In this study, a remote monitoring app along with text messages did not change AET adherence but improved secondary outcomes, such as frequency of high-cost health care encounters, for women with early-stage breast cancer receiving AET.

## Teaser

This randomized clinical trial assesses whether a remote monitoring app with or without additional weekly text messages improves adherence to adjuvant endocrine therapy compared with enhanced usual care among patients with early-stage breast cancer.

## Introduction

Breast cancer is the most common cancer among women, and most women diagnosed have hormone receptor (HR)–positive tumors.^1^ To reduce cancer recurrence risk and increase survival, treatment for HR-positive tumors typically includes long-term use of adjuvant endocrine therapy (AET) after primary treatment (ie, surgery, radiation, and/or chemotherapy).^2,3^ Despite these benefits, adverse effects (eg, joint pain, decreased libido, and hot flashes) contribute to lower adherence.^4,5,6,7,8,9,10,11,12,13,14^ Monitoring adverse symptoms and adherence with tailored education, especially between clinic visits, could help health care practitioners better manage AET–related symptoms and consequently improve long-term adherence and health outcomes.

Although AET adherence plays an important role in reducing recurrence and mortality among women with HR-positive breast cancer,^15^ few behavioral interventions to date have specifically aimed to improve AET adherence, and none showed statistically significant improvements in long-term adherence (ie, 1 year or longer).^16,17,18,19,20,21,22^ Among these, a recent randomized clinical trial (RCT) of a text messaging intervention among women with early-stage breast cancer also failed to improve AET adherence,^21^ highlighting the challenge of improving long-term AET adherence and the potential need for more personalized multilevel interventions. Growing evidence from rigorous randomized trials shows that remote digital symptom monitoring using patient-reported outcomes for adults receiving systemic cancer treatment can reduce emergency department (ED) visits and improve survival.^23,24,25^ Additionally, tailored text messaging has been shown to increase medication adherence for adults with chronic conditions.^22,26,27,28^ To our knowledge, no large RCTs with extended follow-up have evaluated tailored text messaging combined with remote monitoring for women with early-stage breast cancer receiving AET.

We evaluated a mobile app that captures weekly reported AET adherence and related symptoms with alerts to the oncology team and integration with the patient’s electronic health records, with or without additional tailored text messages. Our study was a rigorous RCT to test the effectiveness of these interventions designed to improve patient-physician communication about AET adherence and related adverse symptoms.

## Methods

We conducted a nonblinded RCT to test the effectiveness of a remote monitoring app with built-in alerts to the oncology team, with or without tailored text messages to participants, among women prescribed AET between November 15, 2018, and June 11, 2021.^29,30^ All procedures were approved by the University of Tennessee Health Science Center institutional review board. Eligible patients provided written consent in person before April 20 (prior to the COVID-19 pandemic) and electronic consent by email and signed using the REDCap eConsent framework after April 2020.^31^ The trial was prospectively registered at ClinicalTrials.gov (NCT03592771). The trial protocol and statistical analysis plan have been described previously,^30^ and the protocol is provided in Supplement 1. This study followed the Consolidated Standards of Reporting Trials (CONSORT) reporting guideline for RCTs.^32^

### Participants

Eligible participants were adult women with a diagnosis of ductal carcinoma in situ or stage I to III HR-positive breast cancer and a new prescription for AET, including aromatase inhibitors or tamoxifen, who were treated at a large community comprehensive oncology center serving western Tennessee, northern Mississippi, and eastern Arkansas, with a network of 14 clinic locations providing fully integrated, multidisciplinary cancer care. Eligible patients needed to have a mobile device with a data plan and an email address and be willing to use the electronic pillbox and complete brief surveys. Patients were excluded if they had previously used AET or had rheumatoid arthritis, fibromyalgia, or chronic pain disorder. We also excluded patients concurrently undergoing surgery or chemotherapy or who were unable to communicate in English.

Patients were identified using the electronic health record system and by physician referral. Patients who consented and completed the enrollment survey were randomized using race-stratified (White or other race, based on what was entered in the medical record) block randomization with block size of 6 into 1 of 3 groups—app, app plus feedback, or enhanced usual care (EUC)—by the coordinator (A.N.C. randomized consented participants using REDCap). Race stratification was performed to ensure balance between groups.

### Intervention

#### EUC

All patients received education about AET and treatment-related symptoms. Patients were encouraged to have a follow-up appointment with their oncology team 8 to 12 weeks after initiating AET. At each clinic visit, patients were asked to complete an electronic tablet–based comprehensive symptom screening tool.^33,34^ Between visits, patients could report troublesome symptoms and receive support by calling the clinic.

#### App

In addition to EUC, app group participants received a weekly text message reminder to log into the study app to report adherence and any new or changing symptoms in the prior 7 days. The development process and app design have been described in prior publications.^29,30^ Responses that reached specific thresholds for adherence (3 or more missed doses in the past week) and symptoms (severity of 7 points or higher or increase of 4 points or more from last report, based on a 0-10 scale) triggered an alert to the oncology team. All responses entered in the app were integrated into the patient’s electronic health record system in real time and available for review by the oncology team.

#### App Plus Feedback

In addition to the app, participants in the app plus feedback group received additional weekly tailored text messages, including educational information and encouragement related to AET adherence, AET-related symptoms, lifestyle, social support, and patient-physician communication. Content was tailored to the participant’s race and/or ethnicity, preferred activities and hobbies, sexual identity, and religion reported at enrollment and low-severity symptoms reported in the study app.

### Data and Measures

At enrollment, participants self-reported sociodemographic characteristics, including race and ethnicity, educational level, household income, health literacy, and other patient-reported outcomes (described as follows).^35^ Race and ethnicity were ascertained by self-report. Race categories included American Indian or Alaska Native, Asian, Black or African American, Native Hawaiian or Other Pacific Islander, White, or other (including multiracial), and ethnicity categories were Hispanic or not Hispanic. A 1-year survey was also collected to assess changes in patient-reported outcomes.

Adherence to AET was measured by an electronically monitored pillbox (Wisepill RT300, Wisepill Technologies) and by self-report in the 1-year follow-up survey. The pillbox used mobile telephone technology to wirelessly transmit data each time the device was opened^30^ and was used by all participants for 1 year. From the pillbox data, we created an adherence measure of the proportion of days the participants took their medication according to the prescribed frequency over the 1-year follow-up period. Days hospitalized and prescriber-advised medication pauses were deducted from the denominator. Self-reported AET adherence was assessed with 5 questions regarding how often in the prior 6 months respondents missed an AET dose due to forgetfulness, carelessness, feeling better, feeling worse, or cost. Response options included *often*, *sometimes*, *rarely*, or *never*. We created a dichotomized indicator of higher AET adherence for those who responded *never* or *rarely* to all adherence questions.

App use data were collected from an app data log for app and app plus feedback participants, including app logins and triggered alerts. App use was measured by the number of logins and the total number of triggered alerts among the app and app plus feedback groups during the 6-month intervention.

Symptom management activities were abstracted from patient medical records at 6 months. They included actions by the oncology team to manage AET-related activities, such as temporary AET pauses, discontinuation, changes to a new AET, or a new prescription to manage an AET-related symptom. Participants were categorized as having symptom management if any of these changes were documented in their medical record during the 6-month intervention period.

Symptom burden was collected in the survey using the Functional Assessment of Cancer Therapy–Endocrine Symptoms.^36^ We used individual responses to 5-point Likert scales to calculate a composite score for symptom burden, with higher scores indicating lower symptom burden severity.^37^

Self-efficacy for managing symptoms was collected by survey using the validated 4-item Patient-Reported Outcomes Measurement Information System Self-Efficacy for Managing Symptoms short-form questionnaire.^38^ Participants indicated how confident they were in managing their symptoms on a 5-point scale. Scores ranged from 4 to 20, and higher scores indicate greater self-efficacy for managing symptoms.

Patient-physician communication was collected by survey using the 10-item Communication: Patient and Physician Peer Assessment.^39^ Responses were on a 5-point scale and were averaged over all 10 communication questions.

Quality of life was captured by survey with the 12-Item Short-Form Health Survey,^38,40^ which consists of 12 items with physical component summary and mental component summary scores. The score range is 0 to 100, with higher scores indicating better quality of life.

Health care utilization was measured by survey using questions adapted from the National Health Interview Survey about the number of health care encounters in the prior 6 months, including office visits, ED visits, urgent care visits, and hospitalizations. Because ED visits, urgent care visits, and hospitalizations were relatively rare, we created a combined variable for these high-cost health care encounters. We also created a combined variable for all health care encounters.

### Statistical Analysis

Using results from our group’s pilot trial^41^ and assuming 60% adherence in the EUC group, 75% adherence in the app group, and 85% adherence in the app plus feedback group, we had 95% power to detect a significant difference among the 3 study groups with 240 evaluable participants (80 per group) with a 5% type-1 error rate. We increased the sample size to 300 participants to account for attrition of up to 20%.

For statistical analyses, we described participant characteristics by study group. Among those who completed the 1-year survey, the percentage of missing responses was less than 6.5% across outcomes. Following the intention-to-treat principle, we conducted multiple imputations with chained equations^42^ for missing outcomes due to loss of follow-up or missing responses. Subsequent comparisons and regression results were generated based on the 25 imputed datasets. For AET adherence, we generated mean differences with 95% CIs in the pillbox-captured proportion of days adherent using ordinary least squares regression. Due to the skewed distribution, we also used the quantile regression to compare the median adherence by study arms. Self-reported high adherence was compared by study group using logistic regression. For outcomes measured at enrollment and follow-up, we examined the adjusted difference by study groups controlling for their baseline outcome value. We used ordinary least squares models for app use, app-triggered alerts, symptom burden, quality of life, self-efficacy for managing symptoms, and patient-physician communication. A zero-inflated model was used for health care utilization outcomes due to excessive zeros; zero-inflated Poisson regression was used unless overdispersion was detected, in which case, zero-inflated negative binomial regression was used instead. Sensitivity analyses were conducted among participants who were randomized and completed the 1-year follow-up survey without imputation; the results are provided in eTable 1 in Supplement 2. All data management and analyses were conducted using SAS, version 9.4 (SAS Institute Inc), and Stata, release 16 (StataCorp LLC). Statistical significance was determined using 2-sided tests. Results adjusting for multiple testing with 10 study outcomes (pillbox-monitored adherence, self-reported high adherence, symptom burden, physical health quality of life, mental health quality of life, self-efficacy for managing symptoms, patient-physician communication, office visits, high-cost health care encounters, and all health care encounters) using the conservative Bonferroni adjustment at *P* < .005 were mostly consistent with the findings using a significance threshold of *P* < .05.^43^

## Results

**Figure 1.**
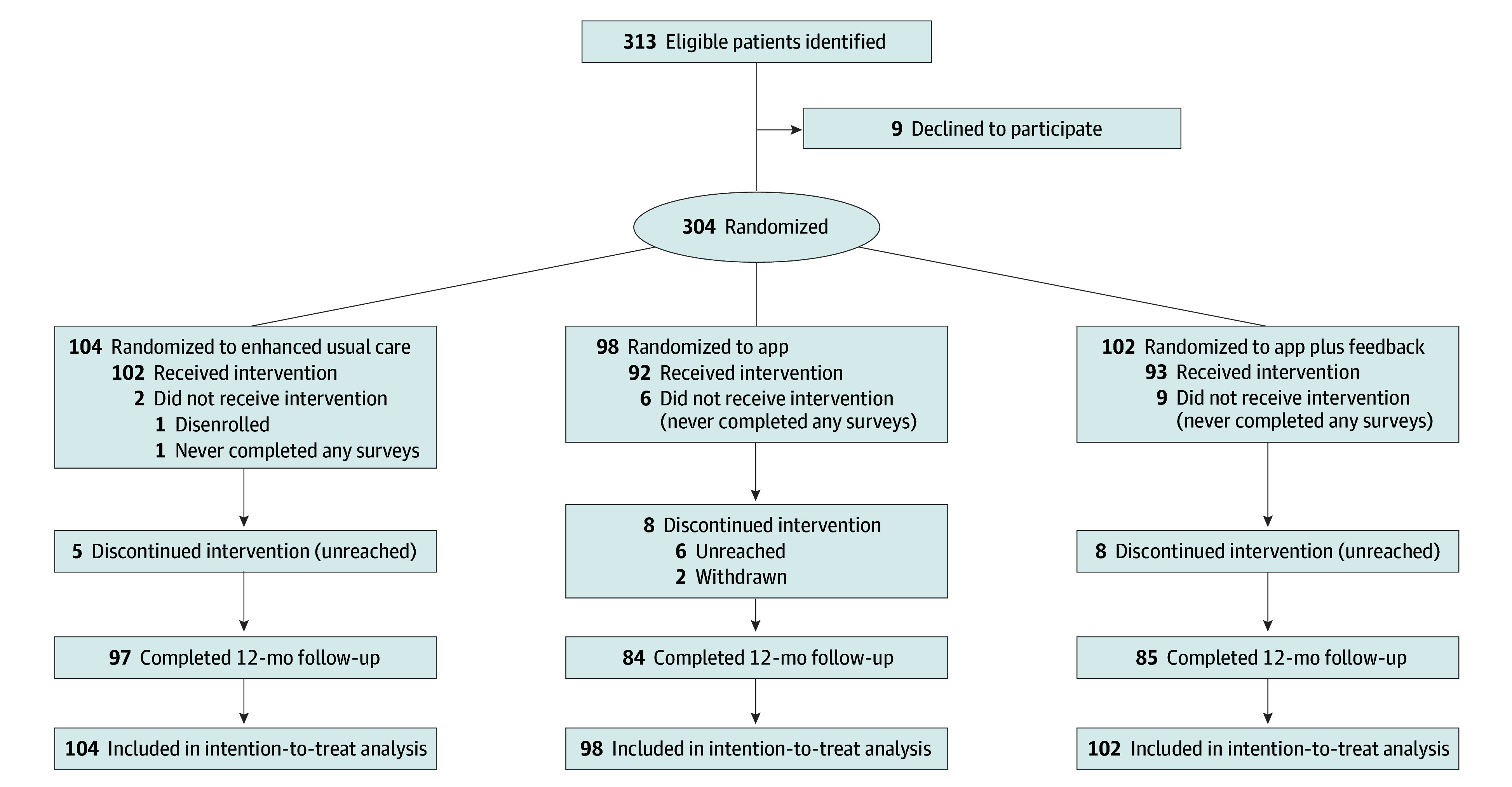
Study Flow Diagram

We identified 313 eligible female patients; 9 (2.9%) declined to participate and 304 (97.1%) consented, completed the enrollment survey, and were randomized (104 to EUC, 98 to the app, and 102 to the app plus feedback). Seventeen patients (5.6%) withdrew or never completed any surveys before starting AET (Figure 1). Overall, 266 participants (87.5% of those randomized) completed the intervention and 1-year follow-up survey.

### Sample Characteristics at Baseline

The mean (SD) participant age was 58.6 (10.8) years; the median age was 60 years (range, 31-83 years). One participant (0.3%) identified as American Indian or Alaska Native, 5 (1.6%) as Asian, 102 (33.6%) as Black or African American, 192 (63.2%) as White, and 4 (1.3%) as multiracial. Nine participants (3.0%) identified as Hispanic ethnicity. Sixty-five (21.4%) had an income below 200% of the federal poverty level, and 60 (19.7%) had an educational level of high school diploma or lower (Table 1).

**Table 1.** Baseline Characteristics

Characteristics	Participants^a^			
	Total (N = 304)	Enhanced usual care (n = 104)	App (n = 98)	App plus feedback (n = 102)
Age, y				
Median (range)	60 (31-83)	61 (31-78)	60 (31-77)	59 (34-83)
Mean (SD)	58.6 (10.8)	58.3 (11.4)	59.4 (10.3)	58.1 (10.7)
Hispanic ethnicity	9 (3.0)	3 (2.9)	3 (3.1)	3 (3.0)
Race				
American Indian or Alaska Native	1 (0.3)	0	1 (1.0)	0
Asian	5 (1.6)	1 (1.0)	2 (2.0)	2 (2.0)
Black or African American	102 (33.6)	36 (34.6)	34 (34.7)	32 (31.4)
Native Hawaiian or Other Pacific Islander	0	0	0	0
White	192 (63.2)	66 (63.5)	58 (59.2)	68 (66.7)
Other	4 (1.3)	1 (1.0)	3 (3.1)	0
Educational level				
High school or less	60 (19.7)	19 (18.3)	18 (18.4)	23 (22.5)
High school diploma or more	244 (80.3)	85 (81.7)	80 (81.6)	79 (77.5)
Income, percentage of FPL				
<100	32 (10.5)	12 (11.5)	13 (13.3)	7 (6.9)
100-200	33 (10.9)	12 (11.5)	9 (9.2)	12 (11.8)
201-400	66 (21.7)	23 (22.1)	20 (20.4)	23 (22.5)
>400	162 (53.3)	52 (50.0)	54 (55.1)	56 (54.9)
Missing	11 (3.6)	5 (4.8)	2 (2.0)	4 (3.9)
Lower health literacy^b^	59 (19.4)	21 (20.2)	20 (20.4)	18 (17.6)
Married or living with a partner	202 (66.4)	72 (69.2)	62 (63.3)	68 (66.7)
Location^c^				
Rural or suburban	74 (24.3)	33 (31.7)	16 (16.3)	25 (24.5)
Urban	230 (75.7)	71 (68.3)	82 (83.7)	77 (75.5)
Initial AET prescription				
Tamoxifen	73 (24.0)	24 (23.1)	22 (22.5)	27 (26.5)
Anastrozole	204 (67.1)	72 (69.2)	70 (71.4)	62 (60.8)
Exemestane or letrozole	27 (8.9)	8 (7.7)	6 (6.1)	13 (12.7)
Cancer stage at diagnosis				
DCIS	34 (11.2)	11 (10.6)	9 (9.2)	14 (13.7)
I	215 (70.7)	73 (70.2)	73 (74.5)	69 (67.6)
II-III	55 (18.1)	20 (19.2)	16 (16.3)	19 (18.6)
Prior chemotherapy	85 (28.0)	23 (22.1)	36 (36.7)	26 (25.5)
Prior radiation	188 (61.8)	63 (60.6)	60 (61.2)	65 (63.7)

Abbreviations: AET, adjuvant endocrine therapy; DCIS, ductal carcinoma in situ; FPL, federal poverty level.

^a^ Data are presented as the No. (%) of participants unless otherwise indicated.

^b^ Participants who reported never, rarely, sometimes, or often feeling confident filling out medical forms by oneself in the enrollment survey were categorized as having lower health literacy, whereas those who reported always feeling confident filling out medical forms by oneself were categorized as having higher health literacy.

^c^ Rural-urban commuting area (RUCA) codes were used to categorize residential location as urban if RUCA was 1 and rural or suburban if RUCA was 2 to 10.

### App Use and Alerts

During the 6-month intervention, the mean number of logins was 18.9 (11.0) for app participants and 16.4 (10.3) for app plus feedback participants (mean difference, 2.49 logins; 95% CI, −0.54 to 5.52 logins; *P* = .11) (eFigure in Supplement 2). App use resulted in a mean (SD) of 2.0 (8.5) symptom alerts among app and 2.0 (5.9) among app plus feedback participants (mean difference, 0.02 alerts; 95% CI, −2.02 to 2.06 alerts; *P* = .98) and 1.7 (3.9) missed-dose alerts among app and 0.8 (1.8) among app plus feedback participants (mean difference, 0.85 alerts; 95% CI, 0.01-1.69 alerts; *P* = .048).

### AET Adherence

**Figure 2.**
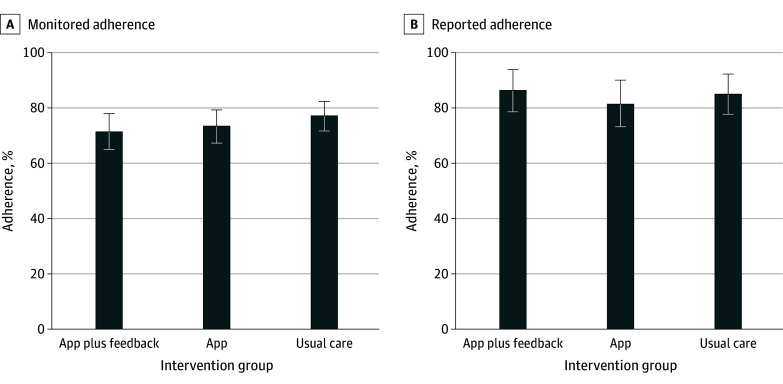
1-Year Adjuvant Endocrine Therapy Adherence Between Intervention Groups Error bars indicate 95% CIs.

Adherence to AET over 1 year measured using the pillbox was similar across groups: 76.6% for EUC, 73.4% for the app (difference vs EUC, −3.3%; 95% CI, −11.4% to 4.9%; *P* = .43), and 70.9% for the app plus feedback (difference vs EUC, −5.7%; 95% CI, −13.8% to 2.4%; *P* = .17) (Figure 2). Self-reported AET adherence at the 1-year follow-up was also similar across groups (EUC, 84.3%; app, 81.0% [difference vs EUC, −3.3%; 95% CI, −14.3% to 7.7%; *P* = .56]; app plus feedback, 85.2% [difference vs EUC, 0.9%; 95% CI, −9.7% to 11.5%; *P* = .87]) (Figure 2).

### Health Care Utilization

Compared with EUC participants, after adjusting for baseline health care utilization levels, app participants did not have significantly fewer health care encounters over the previous 6 months (adjusted difference, −0.65; 95% CI, −1.36 to 0.06; *P* = .07), but app plus feedback participants did (adjusted difference, −1.23; 95% CI, −2.03 to −0.43; *P* = .003) (Table 2). Compared with EUC participants, app participants also did not have significantly fewer high-cost health care encounters at the 1-year follow-up (adjusted difference, −0.27; 95% CI, −0.57 to 0.02; *P* = .07), but app plus feedback participants did (adjusted difference, −0.40; 95% CI, −0.67 to −0.14; *P* = .003). Compared with EUC participants, app participants also did not have fewer office visits at 1-year follow-up (adjusted difference, −0.34; 95% CI, −1.10 to 0.43; *P* = .39), but app plus feedback participants did (adjusted difference, −0.82; 95% CI, −1.54 to −0.09; *P* = .03).

**Table 2.** Adjusted Changes in Secondary Outcomes From Baseline to 1-Year Follow-Up for Intervention Groups Compared With Enhanced Usual Care^a^

Outcome	App		App plus feedback	
	Adjusted difference (95% CI) (N = 304)	*P* value	Adjusted difference (95% CI) (N = 304)	*P* value
Health care encounters in the prior 6 mo				
All health care encounters	−0.65 (−1.36 to 0.06)	.07	−1.23 (−2.03 to −0.43)^b^	.003
High-cost health care encounters^c^	−0.27 (−0.57 to 0.02)	.07	−0.40 (−0.67 to −0.14)^b^	.003
Office visits	−0.34 (−1.10 to 0.43)	.39	−0.82 (−1.54 to −0.09)^b^	.03
Quality of life				
Physical health	1.86 (−0.68 to 4.39)	.15	0.59 (−1.71 to 2.89)	.61
Mental health	−1.73 (−4.30 to 0.84)	.18	0.36 (−2.23 to 2.95)	.78
Other self-reported outcomes				
Symptom burden	−1.20 (−3.87 to 1.47)	.38	0.11 (−2.57 to 2.80)	.93
Self-efficacy for managing symptoms	−0.63 (−1.46 to 0.20)	.13	−0.20 (−1.04 to 0.64)	.64
Patient-physician communication	0.01 (−0.16 to 0.19)	.90	0.13 (−0.04 to 0.30)	.14

^a^ Multiple imputations with chained equations were used for missing outcomes due to loss of follow-up or missing responses. For each outcome, the predictive mean matching method was used, drawing 5 nearest neighbors based on study arm, age, race, educational level, health literacy, marital status, rurality, income level, cancer stage, cancer treatment (receipt of chemotherapy or radiotherapy), and endocrine therapy medication type for imputed values. Multiple imputation was repeated 25 times. Differences were adjusted for baseline values of the corresponding outcome.

^c^ *P* < .05.

^b^ High-cost encounters included emergency department visits, urgent care visits, and hospitalizations.

### Symptom Burden, Quality of Life, Self-Efficacy, and Patient-Physician Communication

We did not find any statistically significant differences in changes over time by study group for symptom burden, quality-of-life scores, and patient-physician communication (Table 2 and eTables 1 and 2 in Supplement 2).

### Symptom Management

During the 6-month intervention, a higher proportion of patients received symptom management in the app plus feedback group (47 of 102 [46.1%]) compared with 36 of 98 (36.7%) app only and 31 of 104 (29.8%) EUC participants. The most common management strategy was adding a new prescription medication to manage an AET-related symptom.

## Discussion

Among patients with early-stage breast cancer who were prescribed AET, an app-based intervention with weekly symptom and adherence monitoring and alerts to the care team, with or without tailored educational text messages, did not improve AET adherence. Nonetheless, with additional tailored messages, the remote monitoring intervention resulted in fewer high-cost health care encounters (ie, hospitalization, ED, and urgent care visits) without increasing the number of office visits compared with EUC.

Although several studies have tested interventions aimed at improving AET adherence, a systematic review found that no prior large-scale behavioral interventions were associated with improved adherence with 1 year or longer follow-up.^44^ Similar to our group’s pilot study,^41^ a few studies have found short-term improvements in adherence.^22,28,45,46^ It is important to note that the adherence rate in the EUC group was higher than the expected rate used for sample size calculations,^30^ which may have limited our ability to detect significant differences between groups. It is possible that giving all participants the electronic pillbox with real-time remote monitoring, which we did not do in the pilot trial, may have increased adherence in the EUC group via the Hawthorne effect.^47^ Our findings highlight the difficulties of increasing long-term AET adherence and the need for novel multilevel sustained approaches that more effectively address adherence barriers.

It is notable that while decreasing the number of office visits, the app plus feedback intervention resulted in a decrease in the number of high-cost health care encounters compared with EUC. Moreover, more frequent remote symptom monitoring did not increase the number of office visits, and with additional tailored messages, it led to fewer office visits, although the *P* value did not reach the conservative Bonferroni adjusted threshold. App-only participants had consistent decreases in health care utilization compared with EUC participants, but these changes were relatively smaller than those of the app plus feedback group and did not reach the threshold of statistical significance. While the magnitude of the reduction in high-cost encounters associated with the app plus feedback intervention may seem modest (0.40 fewer encounters), these events (hospitalizations, ED visits, and urgent care visits) are not common but are highly consequential for patients, health systems, and payers. Consistent with our results, a prior randomized trial of routine symptom monitoring during outpatient chemotherapy for various cancer types also found reductions in ED visits and hospitalizations.^24^ Also consistent with our results, prior trials of remote symptom monitoring alone among patients with early-stage breast cancer had similar trends toward fewer high-cost encounters but did not significantly reduce the number of ED visits.^48,49,50^ Our study extends the prior evidence to show that remote symptom monitoring with additional tailored text messages led to fewer health care encounters, particularly high-cost encounters, without any detrimental impact on quality of life or increasing the number of clinic visits. These findings suggest that most symptoms triggering alerts were resolved with a simple call to a nurse, avoiding additional clinic visits or downstream costly health care encounters.

A possible mechanism for greater reductions in health care utilization among app plus feedback group compared to enhanced usual care may be more active symptom management by the oncology team from alerts and tailored educational messages. During the 6-month intervention, app plus feedback participants had fewer missed-dose alerts compared with app-only participants and more symptom-management changes by their oncology team compared with EUC participants. Our results showing fewer missed-dose alerts and more active symptom management among app plus feedback participants add to prior research showing that tailored messaging combined with remote monitoring was associated with improved care engagement and self-management for patients with early-stage breast cancer.^26,27^

### Strengths and Limitations

This trial had many strengths, including a rigorous randomized study design, racially and economically diverse participants, and 2 measures of medication adherence—electronically monitored and self-reported. Our study also has some limitations. Patients were recruited from a single large, comprehensive cancer center that had already been routinely monitoring patient-reported symptoms at every clinic visit; therefore, our results may not be generalizable to other care settings. For example, we did not detect statistically significant changes in patient-physician communication, self-efficacy for managing symptoms, or quality-of-life composite scores, which were high at enrollment, from the app and app plus feedback interventions. It is possible that other care settings that do not already routinely monitor patient-reported symptoms would have more room for improvement and could experience different results. We limited recruitment to only English-speaking patients with access to an internet-connected computer or smartphone; future research should explore providing symptom monitoring in multiple languages as well as investigate solutions for individuals who do not have technology access. The intervention period was relatively short, only 6 months, for a medication that is usually recommended to continue for 5 or more years. Our recruitment period spanned the start of the COVID-19 pandemic, which caused significant disruptions in care, including fewer in-person visits.^51^ Nonetheless, such disruptions should be similar across study groups, so differences observed by study groups may be attributable to the randomized intervention. Last, we did not collect data on the reason for each health care encounter or whether encounters were related to the patient’s cancer diagnosis or AET use. In future analyses, we plan to evaluate potentially differential effects among subgroups, such as those with lower health literacy or younger ages.

## Conclusions

This RCT found that a remote monitoring app with alerts to the patient’s care team and tailored text messages did not improve AET adherence; however, it reduced overall and high-cost health care encounters without impacting quality of life or increasing the number of office visits. While a prior study found similar reductions in high-cost encounters attributable to weekly symptom monitoring among patients with metastatic tumors receiving systemic treatment,^24^ ours is among the first to show similar benefits of remote monitoring, when combined with tailored text messages, for patients with early-stage breast cancer receiving AET.

## Notes

Supplement 1.
Trial Protocol

Supplement 2. **eFigure.** App Use and Alerts During the 6-Month Intervention Among App and App Plus Feedback Participants **eTable 1.** Adjusted 1-Year AET Adherence and Changes in Secondary Outcomes from Baseline to 1-Year Follow-Up for Intervention Groups Compared With Enhance Usual Care Without Imputation **eTable 2.** Descriptive Outcomes at Enrollment and 1-Year Follow-Up by Randomization Arm With and Without Imputation for Missing Outcomes

Supplement 3.
Data Sharing Statement
