# Supplementary material for: Remote Monitoring App for Endocrine Therapy Adherence Among Patients With Early-Stage Breast Cancer: A Randomized Clinical Trial
Source: JAMA Netw Open. 2024 Jun 27;7(6):e2417873. doi: 10.1001/jamanetworkopen.2024.17873 (PMC11211959; doi:10.1001/jamanetworkopen.2024.17873)
Supplement: Supplement 1. — Trial Protocol [file jamanetwopen-e2417873-s001.pdf]

THRIVE Study Protocol: A randomized controlled trial evaluating a web-based app and tailored messages to improve adherence to adjuvant endocrine therapy among women with breast cancer

Principal investigator. Ilana Graetz, PhD

Trial registration. NCT03592771. Prospectively registered on July 19, 2018.

Protocol Date. August 21, 2019

Protocol publication: Paladino AJ, Anderson JN, Krukowski RA, Waters T, Kocak M, Graff C, Blue R, Jones TN, Buzaglo J, Vidal G, Schwartzberg L, Graetz I. THRIVE study protocol: a randomized controlled trial evaluating a web-based app and tailored messages to improve adherence to adjuvant endocrine therapy among women with breast cancer. BMC Health Serv Res. 2019 Dec 19;19(1):977. doi: 10.1186/s12913-019-4588-x. PMID: 31856812; PMCID: PMC6924011. https://bmchealthservres.biomedcentral.com/articles/10.1186/s12913-019-4588-x#citeas

THRIVE is a five-year study funded by the National Cancer Institute. During the first year, we completed five focus groups to refine the study protocol and app (1). In the second year, we launched the randomized controlled trial of a web-based app and tailored messages for women with breast cancer initiating AET. The intervention lasts 6 months, and the primary end point is medication adherence assessed 12-months after enrollment.

Study Objectives:

1. Test if the App and App+Feedback conditions improve AET adherence.
2. Test if the App and App+Feedback conditions improve symptom burden, quality of life, patient-provider communication, and self-efficacy for managing symptoms.
3. Calculate the relative impact of the App and App+Feedback conditions on healthcare utilization and cost.

Study Setting. Study participants will be enrolled at the West Cancer Center Research Institute (WCCRI). The WCCRI is the largest comprehensive oncology center in the tristate area of West Tennessee, Northern Mississippi, and East Arkansas, with a network of 14 clinic locations providing fully integrated cancer care. The WCCRI treats more than 1,200 patients with a new breast cancer diagnosis each year. The WCCRI serves a diverse patient population similar to the surrounding region: nearly 40% of patients identify as racial/ethnic minorities, the majority of whom identify as Black.

Participants: Potentially eligible patients from all WCCRI locations are identified by our research nurse using WCCRI's EHR system and by physician referral. Patients referred to the study meet with a research nurse who confirms eligibility, obtains informed consent, provides the electronic pillbox device, and has participants complete a baseline survey. Between November 2018 and March 2021, 300 participants will be recruited; participants complete study tasks for a minimum of a year (the primary study end-point), and up to 36 months, depending on how early in the trial they were enrolled. This study received approval from the University of Tennessee Health Science Center Institutional Review Board (IRB #: 17-05479-XP IAA).

Inclusion/exclusion criteria. The criteria for entry into the study include: a) adult female WCCRI patients (ages 18 years and older) with a diagnosis of ductal carcinoma in situ or Stage I-III hormone receptor-positive breast cancer; b) new prescription for an aromatase inhibitor (AI) or tamoxifen; c) have a mobile device with a data plan; d) have a valid email address; e) willing to complete brief surveys on a web-enabled device. Given that side effects associated with AET are typically more severe when treatment is first initiated, we exclude patients who had prior AET use. Because of potential exacerbations of possible side-effect, we also exclude patients with a current diagnosis of rheumatoid arthritis or fibromyalgia. We also exclude patients with chronic narcotic usage. Further, we do not include participants concurrently undergoing surgery or chemotherapy so that we could best disentangle the source of the side effects caused by AET alone. Our survey and app were only available in English, thus we excluded participants who were unable to communicate in English. Aside from chemotherapy and surgery, participants are permitted to undergo radiation and receive other concomitant treatments.

Procedures. Following informed consent, all participants complete a baseline survey using the REDCap (Research Electronic Data Capture) database assessing basic demographic information and baseline measures of key study outcomes (see Table 2). REDCap is a secure web-based application that has many useful features for creating and managing online research databases and surveys while ensuring data integrity, such as auditing trails, and secure data import and export functions (2). The study statistician (MK) generated the randomization sequence with SAS using race-stratified block randomization with equal allocation 1:1:1 with block size of six. Randomization will be implemented in the RedCap protocol database. Only the study statistician and the database manager have access to the randomization scheme. The study coordinator will randomize participants into one of the three study arms: 1) App, 2) App+Feedback, or 3) Usual Care. Finally, the study research nurse orients participants to their assigned condition and provides new enrollees with the study materials for their condition. All participants are given an electronic pill monitor (i.e., a WisePill device) and asked to use it for 12 months. Patients are asked to use this device exclusively with their prescribed AET medication. At enrollment, participants are

trained on the use of the pillbox device, including refill instructions. If participants do not use the WisePill device for 14 consecutive days, study staff contact the participant via text or e-mail to troubleshoot barriers to device use. At the 12-month visit, participants are asked to return their pillbox monitors either in person or using a preaddressed envelope, which is mailed to them. After returning their pill monitors at the end of month 12, participants are given a financial incentive ($60) to compensate them for the time and effort required to use the WisePill monitor. Figure 1 is a flow diagram of patient enrollment, randomization, and assessments through the trial. Additionally, participants are emailed a secure link and asked to complete brief follow-up surveys every six months for the duration of the trial.

Figure 1. THRIVE Study schema

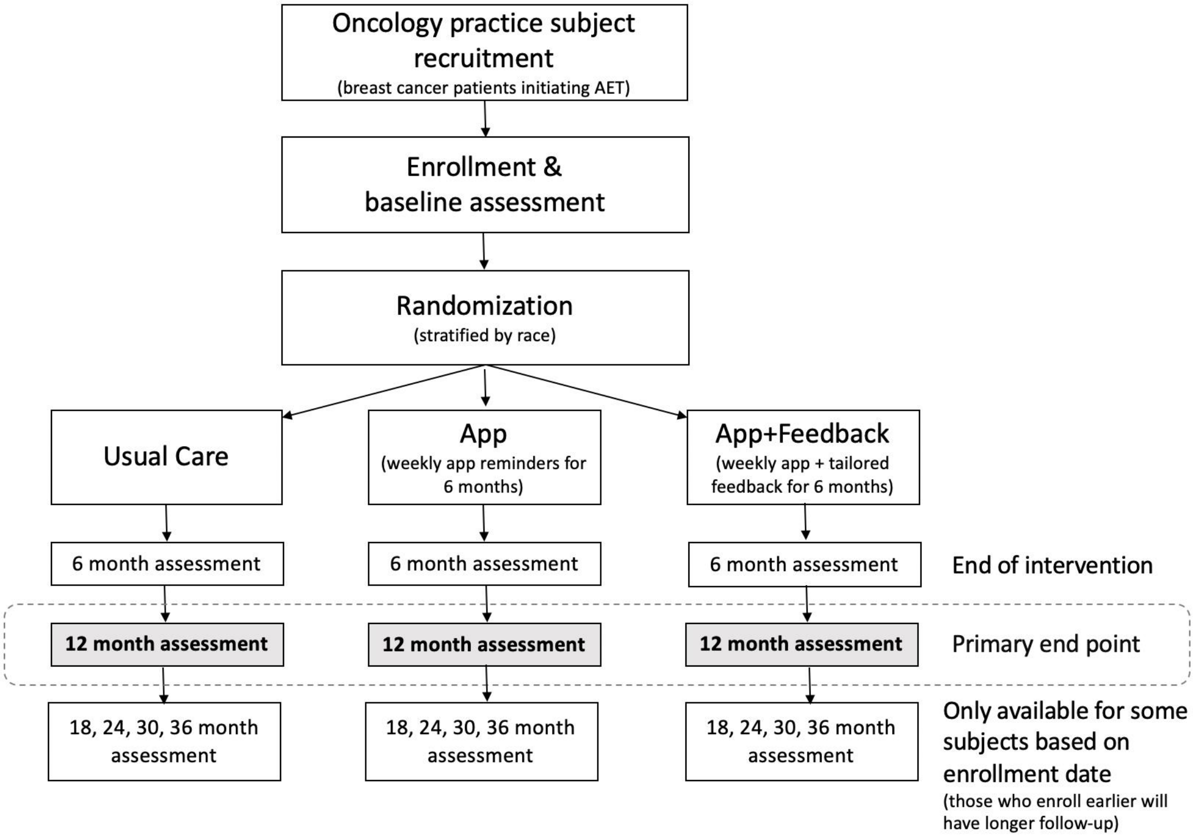

Usual care arm: All patients received standard education about AET treatment and treatment-related symptoms. Patients were encouraged to have a follow-up appointment with their oncology team 8-12 weeks after initiating AET. At each clinic visit, all patients treated at WCCRI are asked to complete the standard WCCRI tablet-based comprehensive symptom screening tool system at each appointment as part of their standard care. Usual Care participants will be asked to use the WisePill box with their AET for 12 months, and complete the surveys at enrollment, 6-months, and 12-months post-enrollment.

App Condition. Participants in the App group receive a weekly text message to prompt them to log into the THRIVE app to answer questions about their AET adherence and related adverse symptoms. The app can be accessed through any web-enabled device or Internet browser. The reporting of medication use is assessed by a single-item adherence measure adapted from the Medication Adherence Reasons Scale (3, 4). Symptoms are assessed using a condensed version of the FACT-ES (5), with follow-up questions about severity of symptoms using a 10-point severity scale. If participants select “Pain” as a symptom, they are prompted with a body map where they can indicate the specific area of the body in which they are experiencing pain. The questions are designed to limit the time burden on patients; reports should take about one to two minutes to complete.

During the formative phase of the study, we conducted focus groups with Black and White women with breast cancer to determine the optimal frequency (e.g., weekly, every other week, or monthly) and timing of messages and content (e.g., motivations, app use, AET adherence, symptom summary, relevant educational materials) (1). These focus groups served to inform our intervention design, primarily through presentation of content to participants and allowing them to rate and provide feedback on both quality of content, utility of certain features, design aesthetic, and other methodological considerations. Examples of some of these findings include preferences for once-a-week reminder messages; a loss aversion scheme for incentivization; and the free text, body map, and patient dashboard features. Although participants are prompted to use the app at least once per week, they are informed that they may use it at any time regardless of when they receive prompts. All patient-reported data are automatically entered into the EHR system and easily available to oncology care teams for review. Furthermore, participants can use the free-text feature to convey any information they want to share with their provider that is not captured in the questions asked in the app.

The app alerts are based on response thresholds to adherence and symptom questions and are generated to inform the participant's care team (i.e., prescribing physician and nurse) of any concerning responses or trends that emerge from the participant-reported outcomes via the app. WCCRI oncologists guided the alert thresholds, which include: three missed doses within the last week, a 4-point increase in symptom burden on the severity scale, or a score of 7-10 on the severity scale. Participants are also able to provide a free-text response to report anything they would like at the end of the survey, which is reviewed by the nurse coordinator who determines if a response from the oncology team is required.

These alerts inform providers of potentially concerning symptoms that warrant care team contact with the patient. The alert messages include the event that triggered the alert and are sent to the care team via e-mail. Care teams are asked to respond to alerts within 48 hours. They are able to review concerning responses directly from the patient's EHR to help guide ongoing treatment and make therapeutic adjustments when necessary. AET nonadherence for three days or more within one week prompts a call, even when the cause is not related to an adverse symptom. This facilitates communication between patients and their teams on barriers to AET adherence and provides an opportunity for shared decision-making. A research nurse notes the clinical response and care team contact in the patient's record after each alert (i.e., a phone consult, visit, and any medication changes).

App+Feedback Condition. In addition to the previously outlined procedures, participants randomized to the App+Feedback group will receive weekly tailored feedback messages based on their baseline survey responses and use of the app during the 6-month intervention phase. Some tailored feedback includes links to symptom-specific educational materials and coping strategies for participants who report low-severity symptoms. Using feedback from focus groups, we developed a library of messages with multiple options for each condition in order to prevent desensitization to the same message. Message categories are tailored to participant's responses to the app and baseline survey. Some feedback messages are supplemented with images.

Data Collection

Medication Adherence Monitoring. The WisePill monitor consists of a pillbox that wirelessly transmits adherence data each time the device is opened. The device uses mobile phone and Internet technologies to provide real-time adherence data. WisePill has been used to track medication adherence for other medications used to treat various diseases (6, 7).

Surveys. Participants are asked to complete brief online surveys every 6 months during their participation in the trial. Participants who do not complete the online follow-up surveys are offered other modalities to improve response rates, including mailing a paper version with a self-addressed return envelope or via phone interview with the research nurse. Table 1 describes survey measures collected.

Table 1. Key Survey Measures and Data Collection Timeline

Measure	Description of Measure	Survey Time Points: Baseline	12-month Follow-Up	18+ Month Follow-up
Demographics	Age, highest level of education, total household income, race/ethnicity, current relationship status, sexual orientation, gender identity, and religious identity will be collected at baseline	X		
AET medication Adherence	Self-reported medication adherence will be measured with 5 questions about frequence of adherence and main reasons.		X	X
Symptom burden (FACT-ES)	The Functional Assessment of Cancer Therapy Endocrine Symptoms (FACT-ES), a 18-item instrument evaluates endocrine symptoms on a five-point Likert scale (5)	X	X	X
Quality of life (SF-12)	The Short-Form Health Survey (SF-12), a 12-item instrument that provides summary measures of physical and mental health status will be used (8, 9)	X	X	X
Communication: Patient and Physician Peer Assessment Module;	The Patient and Physician Peer Assessment Module (10) is an 11-item instrument that assesses patient perceptions of communication with their provider on scale of 1 (poor) to 5 (excellent).	X	X	X
Self-efficacy for managing symptoms (PROMIS)	*The 4-item PROMIS Item Bank v1.0* – Self-Efficacy for Managing Symptoms short form scale will be used to measure confidence in a participant's ability to successfully perform specific tasks or behaviors related to her health in a variety of situations (11)	X	X	X
Healthcare utilization in previous 6-months	A measure developed for this study based on the National Health Institutes Survey (NHIS) (12) will be used to assess healthcare utilization throughout the duration of the study.	X	X	X

Electronic Health Record Chart Abstraction. The following baseline demographic data are abstracted from the patient's electronic health record upon enrollment:

- Date of birth, race/ethnicity, marital status, comorbidities
- Disease stage, tumor histology and grade, hormone receptor and human epidermal growth factor-2 (HER-2) status, menopausal status
- AI therapy and modalities of breast cancer treatment received in the primary adjuvant setting
- Chemotherapy and/or surgery received prior to AET
- Provider Responses to the alerts
- Changes in AET including temporary pauses, discontinuations, changes to a different AET, and new prescriotion to manage an AET-related symptoms.

Intervention Fidelity Monitoring and Data Management. We adhere to the following quality procedures to ensure treatment fidelity: 1) development of detailed intervention standard operating procedures; 2) electronic monitoring of receipt of emails/texts, app usage, and feedback reports; 3) documentation of all intervention contacts; and 4) weekly meetings to review overall adherence to structured protocols, and problem solving for any issues related to participant challenges. The study coordinator performs weekly data quality checks, and the study statistician performs range checks for data values on a monthly basis. All identifiable data is stored on password protected servers or locked file cabinets that only the study team has access to. The study PI, the study statistician, and the database manager will have access to the data. Quarterly reports will be generated from the accumulating data and the study team will carefully review the data for missingness and type of missingness and accuracy in data capture, and will develop timely action plans when necessary.

If any safety adverse events are discovered, a safety protocol will be followed according to standardized procedures used by the West Cancer Center as standard of care under the guidance of Drs. Schwartzberg and

Vidal. All unanticipated adverse events will be recorded in a form that includes event date, whether the event is treatment related, and date event was addressed. The form will be given to Dr. Graetz within 24 hours of learning of the event and the event documented by the appropriate staff member in progress notes, and reported to IRB, if appropriate. Alerts, adverse events, and referrals will not cause a participant to be dropped from the study, but will be considered in analysis. Any contact outside planned study contact, will be documented (time, reason, actions taken, initiator) on the Delivery Assessment tracking form

Study Retention. The pilot study retention rate at eight weeks was 88% without the use of financial incentives to compensate patients for their time and contribution to the study. This high rate may reflect the proactive approach that we have employed, including maintaining current contact information, minimizing barriers by offering multiple modalities to complete surveys, and regular study meetings with retention as a standing agenda item. For this study, we are following participants for a longer period, which could impact retention rates; to maintain high retention rates, we provide small but meaningful incentives through a loss aversion scheme, as recommended by participants during the formative research phase. This includes a total compensation up to $220 in merchant credit depending on when they first enroll and how many follow-up surveys they complete. Specifically, for months 0-6, all participants are credited $60 merchant credit at the end of the period if they complete all activities, including the 6-month survey and maintenance of the WisePill device's battery charge. For months 7-12, participants receive an additional $120 merchant credit for the second follow-up survey, WisePill battery charge maintenance, and return of the WisePill device. For months 18-36, participants who enroll early are asked to complete follow-up surveys every 6 months and receive $10 per survey, for a maximum of $40.

Criteria for modifying allocated interventions. If a participant is discontinued from adjuvant therapy, she is asked to still complete the follow-up surveys, but no longer receives reminder or feedback messages nor completes the app surveys if she was allocated to the app or app+feedback conditions. Additionally, participants could voluntarily withdraw from the study at any time.

Statistical Analysis

***Power/Sample Size*.** Using adherence results from our pilot study and assuming 60% adherence in the 'Usual Care' arm, 75% adherence in the 'App' arm, and 85% adherence in the 'App+Feedback' arm, 95% power to detect a significance adherence difference among the three arms will be achieved with a total of 240 evaluable participants (80 evaluable patients in each arm) with 5% Type-1 error rate. We increase the sample size to 100 for each arm for a total accrual of 300 participants to account for potential 1-year attrition up to 20%. The same sample size would also provide 90% power with 5% Type-1 error rate to significantly detect the Quality of Life difference of 9.3 units where the 'App' and 'App+Feedback' arms are combined against the 'Usual Care' arm with projected standard deviations from the pilot study, of 9.3 and 24.3, respectively.

Primary Outcome

Adherence. Using the electronic pill monitoring system data, adherence will be defined as the proportion of days in which each participant took her medication (as recorded and transmitted via WisePill device opening) according to the prescribed frequency during the 12-month study period. For example, a patient would be considered to be 100% adherent if the pill monitoring electronic data showed that the bottle was opened on 365 days. Days during which patients were hospitalized will be deducted from the denominator. The electronic medication monitoring system will be used solely as an outcome measure to compare AET adherence among the study groups, and it will not be used in the app, clinic alerts, or feedback reports.

Secondary Outcomes

Quality of life, patient-provider communication, self-efficacy for managing symptoms, and symptom burden. Descriptive statistics of baseline characteristics on all participants will be presented and compared among the three treatment groups as means and standard deviations for continuous variables, and as frequencies and percentages for categorical variables. One-way analysis of variance (ANOVA) and t-tests for pairwise comparison, or its non-parametric counterparts such as Kruskal-Walls (or Wilcoxon-Mann-Whitney) test when warranted, and chi-squared or Fisher's exact tests will be used to compare the groups for any differences in characteristics. The primary data analysis will adhere to the intent-to-treat principle.

Healthcare utilization and costs. To ensure completeness, we will combine self-reported healthcare

utilization (reported every 6 months) with data abstracted from patient EHRs. Duplicates will be eliminated, and if conflicting information arises, we will assume that the EHR is correct. All utilization will be converted to cost using Medicare reimbursement rates. Using Medicare payment rates is a relatively standard approach in economic analysis because these rates provide a common metric for costing out services across all sectors of care (public or private) (13); this is important because we are actually interested in the underlying resource utilization, not differences in prices. Medicare is also a major payer in all health care markets; almost all payers 'follow Medicare's lead' when determining payment rates. Finally, many Medicare reimbursement rates were originally determined based on cost studies; thus, the Medicare relative fee structure bears some resemblance to the underlying relative cost structure.

For analysis of healthcare utilization data, we will employ two-part Hurdle Poisson models that are appropriate for ''rate'' data (e.g., visits per year). If we find that the data are over-dispersed, we will also explore negative binomial models. These models will yield two sets of coefficients, one related to the probability of any utilization and the other related to the level of utilization conditional on having any utilization. For cost data, we will employ two-part lognormal models to accommodate the high level of skewness. Again, this approach will yield two sets of coefficients: one related to probability of any cost and the other related to level of cost (conditional on having any cost). We have successfully used this approach in previous studies. We will also use survey reports from providers at clinic staff to accurately estimate costs to implement and maintain each intervention. For relative cost-effectiveness, we will calculate 6- and 12-month incremental cost-effectiveness ratios, comparing App or App+Feedback versus usual care at 6 and 12 months.

Missing data. Missing data, especially relating to the primary objective of the study, will be evaluated while the study is ongoing to develop corrective actions if possible. Once the study is completed, the missing data structure will be assessed and appropriate imputation approaches will be implemented if necessary.

List of abbreviations

AET: Adjuvant endocrine therapy
EHR: Electronic health record
WCCRI: West Cancer Center Research Institute

Declarations

Ethics approval and consent to participate

This study protocol (version 1.17 from August 2, 2019) was approved by the University of Tennessee Health Science Center Institutional Review Board (IRB #: 17-05479-XP IAA). We will obtain written informed consent form all study participants.

Funding. This study is supported by a grant from the National Cancer Institute (1R01CA218155). The funding source had no role in the design of this study and will not have any role during its execution, analysis, interpretation of the data, or

References

1. Paladino AJ, Anderson JN, Graff JC, Krukowski RA, Blue R, Jones TN, et al. A qualitative exploration of race-based differences in social support needs of diverse women with breast cancer on adjuvant therapy. Psycho-Oncology. 2019;28(3):570-6.
2. Harris PA, Taylor R, Thielke R, Payne J, Gonzalez N, Conde JG. Research electronic data capture (REDCap)—a metadata-driven methodology and workflow process for providing translational research informatics support. Journal of biomedical informatics. 2009;42(2):377-81.
3. Unni EJ, Olson JL, Farris KB. Revision and validation of Medication Adherence Reasons Scale (MAR-Scale). Current medical research and opinion. 2014;30(2):211-21.
4. Unni E, Farris KB. Determinants of different types of medication non-adherence in cholesterol lowering and asthma maintenance medications: a theoretical approach. Patient education and counseling. 2011;83(3):382-90.
5. Fallowfield LJ, Leaity SK, Howell A, Benson S, Cella D. Assessment of quality of life in women undergoing hormonal therapy for breast cancer: validation of an endocrine symptom subscale for the FACT-B. Breast Cancer Res Treat. 1999;55(2):189-99.
6. Haberer JE, Kiwanuka J, Nansera D, Muzoora C, Hunt PW, So J, et al. Real-time adherence monitoring of antiretroviral therapy among HIV-infected adults and children in rural Uganda. AIDS (London, England). 2013;27(13).
7. Vervloet M, van Dijk L, Santen-Reestman J, van Vlijmen B, Bouvy ML, de Bakker DH. Improving medication adherence in diabetes type 2 patients through Real Time Medication Monitoring: a randomised controlled trial to evaluate the effect of monitoring patients' medication use combined with short message service (SMS) reminders. BMC Health Services Research. 2011;11(1):5.
8. Ware J, Kosinski M, Turner-Bowker DM, Gandek B. User's manual for the SF-12v2 Health Survey. Lincoln, RI: QualityMetric Incorporated. 2002.
9. Ware JE, Keller SD, Kosinski M. SF-12: How to score the SF-12 physical and mental health summary scales: Health Institute, New England Medical Center; 1995.
10. Symons AB, Swanson A, McGuigan D, Orrange S, Akl EA. A tool for self-assessment of communication skills and professionalism in residents. BMC medical education. 2009;9(1):1.
11. Cella D, Riley W, Stone A, Rothrock N, Reeve B, Yount S, et al. The Patient-Reported Outcomes Measurement Information System (PROMIS) developed and tested its first wave of adult self-reported health outcome item banks: 2005–2008. Journal of clinical epidemiology. 2010;63(11):1179-94.
12. Blackwell DL, Lucas JW, Clarke TC. Summary health statistics for US adults: national health interview survey, 2012. Vital and health statistics Series 10, Data from the National Health Survey. 2014(260):1-161.
13. Miller SM, Shoda Y, Hurley K. Applying cognitive-social theory to health-protective behavior: breast self-examination in cancer screening. Psychological bulletin. 1996;119(1):70.
