# Supplementary material for: Remote Monitoring App for Endocrine Therapy Adherence Among Patients With Early-Stage Breast Cancer: A Randomized Clinical Trial
Source: JAMA Netw Open. 2024 Jun 27;7(6):e2417873. doi: 10.1001/jamanetworkopen.2024.17873 (PMC11211959; doi:10.1001/jamanetworkopen.2024.17873)
Supplement: Supplement 2. — eFigure. App Use and Alerts During the 6-Month Intervention Among App and App Plus Feedback Participants eTable 1. Adjusted 1-Year AET Adherence and Changes in Secondary Outcomes from Baseline to 1-Year Follow-Up for Intervention Groups Compared With Enhance Usual Care Without Imputation eTable 2. Descriptive Outcomes at Enrollment and 1-Year Follow-Up by Randomization Arm With and Without Imputation for Missing Outcomes [file jamanetwopen-e2417873-s002.pdf]

Supplementary Online Content

Graetz I, Hu X, Kocak M, et al. Remote monitoring app for endocrine therapy adherence among patients with early-stage breast cancer: a randomized clinical trial. *JAMA Netw Open*. 2024;7(6):e2417873. doi:10.1001/jamanetworkopen.2024.17873

eFigure. App Use and Alerts During the 6-Month Intervention Among App and App Plus Feedback Participants
eTable 1. Adjusted 1-Year AET Adherence and Changes in Secondary Outcomes from Baseline to 1-Year Follow-Up for Intervention Groups Compared With Enhance Usual Care Without Imputation
eTable 2. Descriptive Outcomes at Baseline and 1-Year Follow-Up by Randomization Arm With and Without Imputation for Missing Outcomes

This supplementary material has been provided by the authors to give readers additional information about their work.

eFigure. App Use and Alerts During the 6-month Intervention Among App and App Plus Feedback Groups

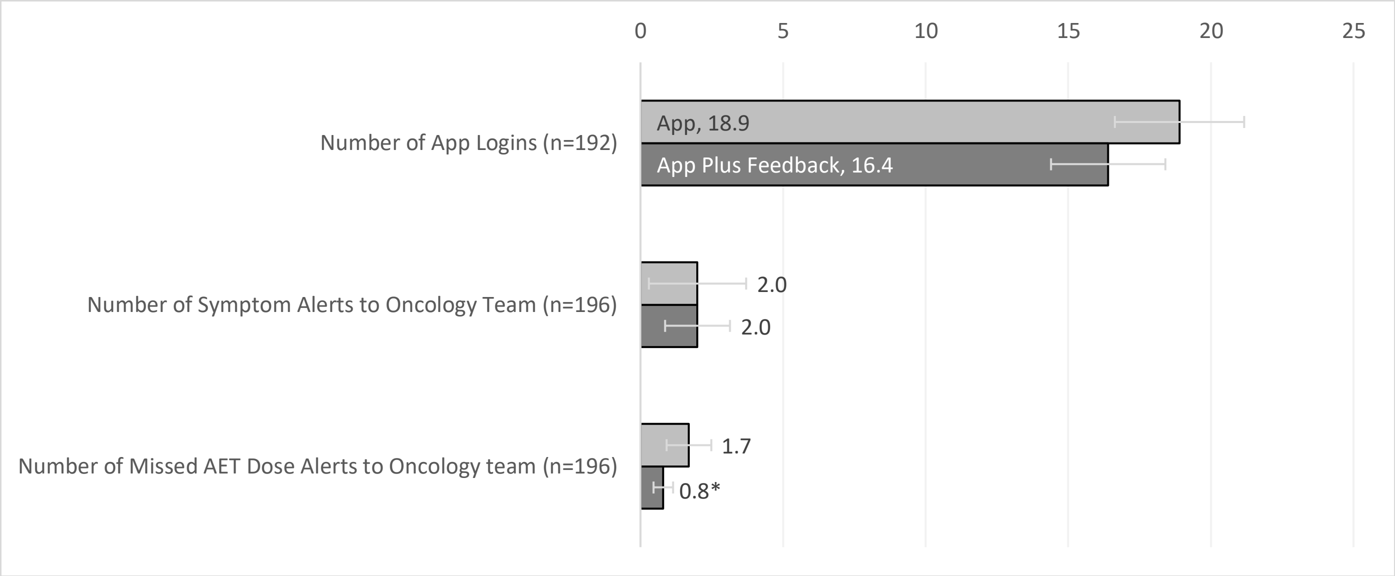

We compared differences in logins and alerts using ordinary least square regression. Margins command used to calculate rates of each outcome by study arm. Error bars indicate 95% CIs.

[a]*P*<0.05.

© 2024 Graetz I et al. *JAMA Network Open*.

eTable 1. Adjusted 1-Year Adjuvant Endocrine Therapy Adherence and Changes in Secondary Outcomes from Baseline to 1-Year Follow-Up for Intervention Groups Compared With Enhance Usual Care Without Imputation

	Adjusted Difference (95% CI)	
**Outcomes**	**App**	**App Plus Feedback**
1-Year adjuvant endocrine therapy adherence		
Monitored adherence (N=266)	-3.7% (-12.0%, 4.5%)	-5.6% (-13.8%, 2.6%)
Reported adherence (N=264)	-3.4% (-14.5%, 7.7%)	1.3% (-9.1% to 11.8%)
Health care encounters in the prior 6 months[a]		
All Healthcare Encounters (N=258)	-0.63 (-1.32, 0.06)	-1.31[c] (-1.98, -0.65)
High-Cost Healthcare Encounters[b] (N=258)	-0.30 (-0.60, 0.00)	-0.41[c] (-0.68, -0.14)
Office Visits (N=258)	-0.21 (-1.29, 0.87)	-0.67 (-1.69, 0.36)
Quality of life[a]		
Physical health (N=253)	2.20 (-0.19, 4.58)	1.07 (-1.32, 3.45)
Mental health (N=253)	-1.89 (-4.40, 0.62)	-0.16 (-2.66, 2.35)
Other self-reported outcomes[a]		
Symptom burden (N=266)	-0.90 (-3.53, 1.73)	0.09 (-2.53, 2.71)
Self-efficacy for managing symptoms (N=253)	-0.58 (-1.42, 0.26)	-0.22 (-1.07, 0.63)
Patient-physician communication (N=264)	0.01 (-0.17, 0.19)	0.12 (-0.05, 0.30)

[a] Differences adjusted for the baseline values of the corresponding outcome.

[b] High-cost encounters included emergency department visits, urgent care visits, and hospitalizations.

[c] $P<0.05$.

© 2024 Graetz I et al. *JAMA Network Open*.

eTable 2. Descriptive Outcomes at Each Timepoint (Baseline and 1-Year Follow-Up) by Randomization Arm With and Without Imputation for Missing Outcomes

	Without Imputation			With Imputation[a]		
	Enhanced Usual Care	App	App Plus Feedback	Enhanced Usual Care	App	App Plus Feedback
	Mean (SE)	Mean (SE)	Mean (SE)	Mean (SE)	Mean (SE)	Mean (SE)
1-Year adjuvant endocrine therapy adherence, %						
Monitored (N=266)	76.6 (2.7)	72.9 (3.0)	71.0 (3.3)	76.6 (2.6)	73.4 (3.0)	70.9 (3.3)
Reported (N=266)	84.4 (3.7)	81.0 (4.3)	85.7 (3.8)	84.3 (3.8)	81.0 (4.2)	85.2 (4.0)
Total health care encounters in the prior 6-months						
Baseline (N=300)	11.75 (1.50)	11.68 (1.38)	12.06 (1.20)	11.67 (1.48)	11.70 (1.38)	12.06 (1.20)
1-year (N=261)	4.78 (0.56)	4.11 (0.48)	3.48 (0.44)	4.79 (0.56)	4.08 (0.45)	3.49 (0.42)
Higher-cost health care encounters in the prior 6-months[b]						
Baseline (N=300)	0.94 (0.16)	1.33 (0.52)	0.82 (0.14)	0.95 (0.19)	1.33 (0.51)	0.82 (0.14)
1-year (N=261)	0.71 (0.15)	0.45 (0.11)	0.30 (0.06)	0.70 (0.15)	0.47 (0.12)	0.29 (0.07)
Office visits in the prior 6-months						
Baseline (N=300)	10.81 (1.48)	10.35 (1.20)	11.24 (1.17)	10.76 (1.46)	10.41 (1.20)	11.24 (1.17)
1-year (N=261)	4.08 (0.54)	3.66 (0.46)	3.18 (0.42)	4.08 (0.54)	3.65 (0.45)	3.17 (0.39)
Quality of life: Physical health						
Baseline (N=301)	44.30 (1.00)	44.87 (0.99)	43.99 (0.96)	44.30 (0.99)	44.74 (1.00)	44.05 (0.96)
1-year (N=255)	44.69 (1.11)	47.57 (1.13)	45.25 (1.15)	44.73 (1.07)	46.88 (1.16)	45.16 (1.08)
Quality of life: Mental health						
Baseline (N=301)	50.66 (0.95)	50.76 (1.00)	50.68 (1.00)	50.60 (0.95)	50.64 (1.01)	50.74 (0.99)
1-year (N=255)	51.77 (0.86)	49.98 (1.18)	51.95 (1.07)	51.42 (0.90)	49.71 (1.12)	51.84 (1.03)
Symptom burden						
Baseline (N=304)	62.95 (0.97)	63.01 (0.97)	63.47 (0.91)	62.95 (0.97)	63.01 (0.97)	63.47 (0.91)
1-year (N=266)	60.14 (1.20)	59.29 (1.25)	60.54 (1.22)	60.15 (1.17)	58.99 (1.22)	60.65 (1.19)
Self-efficacy						
Baseline (N=302)	17.28 (0.29)	16.69 (0.39)	16.88 (0.31)	17.31 (0.28)	16.69 (0.39)	16.88 (0.31)
1-year (N=262)	17.39 (0.28)	16.46 (0.43)	16.78 (0.37)	17.35 (0.28)	16.40 (0.41)	16.93 (0.34)
Patient-physician communication						
Baseline (N=304)	4.66 (0.06)	4.54 (0.07)	4.68 (0.05)	4.66 (0.06)	4.54 (0.07)	4.68 (0.05)
1-year (N=264)	4.57 (0.07)	4.52 (0.08)	4.69 (0.05)	4.56 (0.07)	4.53 (0.08)	4.70 (0.05)

[a] Sample size with imputation is 304. We used multiple imputations with chained equations due to loss of follow-up or missing responses. For each outcome, we used the predictive mean matching method, drawing 5 nearest neighbors based on study arm, age, race, education, health literacy, marital status, rurality, income level, cancer stage, cancer treatment (receipt of chemotherapy or radiotherapy), and AET medication type for imputed values. Multiple imputation was repeated 25 times.

[b] High-cost encounters included emergency department, urgent care, and hospitalizations

© 2024 Graetz I et al. *JAMA Network Open*.
