# Supplementary material for: Remote Monitoring App for Endocrine Therapy Adherence Among Patients With Early-Stage Breast Cancer: A Randomized Clinical Trial
Source: JAMA Netw Open. 2024 Jun 27;7(6):e2417873. doi: 10.1001/jamanetworkopen.2024.17873 (PMC11211959; doi:10.1001/jamanetworkopen.2024.17873)
Supplement: Supplement 3. — Data Sharing Statement [file jamanetwopen-e2417873-s003.pdf]

Data Sharing Statement

Graetz. Remote Monitoring App for Endocrine Therapy Adherence Among Patients With Early-Stage Breast Cancer. *JAMA Netw Open*. Published June 27, 2024.
doi:10.1001/jamanetworkopen.2024.17873

Data
Data available: No
